# Metabolic and Transcriptomic Responses of Weaned Pigs Induced by Different Dietary Amylose and Amylopectin Ratio

**DOI:** 10.1371/journal.pone.0015110

**Published:** 2010-11-30

**Authors:** He Jun, Chen Daiwen, Yu Bing

**Affiliations:** 1 Institute of Animal Nutrition, Sichuan Agricultural University, Ya'an, Sichuan, People's Republic of China; 2 Key Laboratory of Animal Disease-Resistance Nutrition, Sichuan Agricultural University, Ya'an, Sichuan, People's Republic of China; New Mexico State University, United States of America

## Abstract

Starch is one of the major dietary energy sources for mammals. However, the nutritional value of starch largely depends on its amylose and amylopectin ratio. In this study, the overall metabolic and transcriptomic responses of weaned pigs fed with different dietary starches were assessed. Sixteen weaned pigs were randomly allotted to two experimental diets containing either of pure cassava starch (CS) or maize starch (MS) as the sole energy source (the amylose-amylopectin ratio were 0.25 and 0.43, respectively). Results indicated that the body weight gain was not affected by different dietary starches. However, a moderate fatty liver was observed in CS-fed group. Long-term ingestion of CS not only increased the total liver fat content, but significantly elevated the liver triglyceride and cholesterol content (*P*<0.05). In addition, the serum insulin and cholesterol concentrations were both elevated in CS-fed group (*P*<0.05). Microarray analysis led to the identification of 648 genes differentially expressed in liver (*P*<0.05), and a lot of them were involved in lipid and carbohydrate metabolism. Additionally, pathway analysis indicated that both the insulin and PPAR signaling pathways were acutely affected by dietary amylose-amylopectin ratio. Long-term ingestion of CS activated the transcription of lipogenic genes such as *hmgr* and *fasn*, but decreased the expression of lipolytic genes such as *aox1*, *ppara* and *fbp*. The microarray results correlated well with the measurements of several key enzymes involved in hepatic lipid metabolism. Our results suggested that both the metabolic and transcriptomic responses of weaned pigs were tightly regulated by dietary starch composition, and a high amylose ratio starch (i.e MS) may be more healthful for mammals as the long-term energy source by down-regulation of hepatic lipogenesis and steroidogenesis.

## Introduction

Obesity and obesity-associated fatty liver disease (FLD) are becoming global health problems in adults as well as children [1]. FLD is a metabolic disease characterized by large vacuoles of triglyceride fat accumulating in liver cells via the process of steatosis. It has long been documented that dietary energy intake plays a central role in FLD formation. If the consumption of energy far exceeds the combustion of calories, the unburnt energy is conserved in the form of fat in adipose tissue and liver, leading to obesity and FLD [2], [3]. Previous studies also indicated that the hepatic *de novo* lipid synthesis from carbohydrate remains quantitatively small under conditions of energy balance. It can, however, be markedly stimulated by intake of extra carbohydrates or by acute ethanol administration [4]–[7]. Furthermore, the composition of dietary carbohydrates also seems to affect hepatic metabolisms in different ways and to varying degrees [2], [8], [9].

Being diagnosed with FLD, patients are often advised to adjust their dietary energy source to avoid intake of excess energy. Basically, it was generally believed that the carbohydrates might be more healthful than lipids as a daily energy supplier [10]. However, the recommendation of a low-fat-high-carbohydrate diet may even promote the consumption of sugar and highly refined carbohydrates which may lead to obesity and dyslipidaemia, especially among insulin-resistant individuals [11]–[13]. The starch, acting as the major energy-producing component of the daily diet, is the main carbohydrate in mammal nutrition. However, the nutritional value of starches can vary widely depending upon its sources (ie. grain or forage) and sites (ie. rumen, small intestine or large intestine) of digestion [14]. More recently, it was reported that the metabolic responses and health effects induced by different dietary starches may significantly differ [15], [16]. For instance, starches with a high amount of amylopectin are easily digested, which may lead to a rapid and stronger glycaemic and insulinaemic responses [2], [17]. Although these physiological and metabolic responses were previously observed both in human and other mammals [15], [17], [18], little is still known about the mechanisms behind these responses.

In recent years, DNA microarray technology has been widely used to discover gene's functions, to understand biochemical pathways and to discover drug targets [19]. Because of its similar homology to human, the pigs have been recognized as one of the ideal models for the study of clinic nutrition. Studying the profiles of hepatic gene expression in pigs may provide the first information on those diet-induced metabolic responses. Moreover, a better understanding of the starch nutrition is also necessary for people to make intelligent food choices and avoid liver diseases. Therefore, the aim of this study was to assess the overall metabolic and transcriptomic responses of weaned pigs in response to different dietary starches (with different amylose-amylopectin ratio). Furthermore, the mechanisms behind these starch-induced responses were investigated on a molecular basis.

## Methods

### Animal models and diets

The experimental protocols used in the present study were approved by Sichuan Agricultural University Institutional Animal Care and Use Committee (2009-09-03). A total of sixteen male pigs [Duroc × (Landrace × Yorkshire)] weaned at 21 d (average initial body weight of 7.37±0.25 kg) were selected and allotted to two dietary treatments on the basis of nearly equalized weight (n = 8). The experimental diets were formulated on the basis of nutrient requirements for 5–10 kg pigs (National Research Council-Nutrient Requirements of Swine) [20]. Either purified cassava starch (CS) or maize starch (MS) was used as the sole dietary energy source. Both the two starches were purchased from Chengdu food market (Chengdu, Sichuan, China), and the amylose-amylopectin ratios were 0.25 and 0.43, respectively. There were no discrepancies for other nutrient components. Dietary amino acids were supplied by dehulled soybean meal, extruded soybean, and fish meal, and vitamin and minerals were supplied by vitamin and mineral supplements (Table 1). Synthetic DL-methionine was added to the diets to meet minimal methionine-cystine requirements.

**Table 1:** Composition of experimental diet (as fed-basis).

Ingredients	% of diet
Cassava or maize starch	54.50%
Dehulled soybean meal	2.00%
Extruded soybean	10.00%
Soy protein concentrate	17.83%
Whey powder	7.30%
Fish meal	6.00%
CaHPO_4_	0.70%
CaCO_3_	0.55%
Salt	0.15%
Choline Chloride (50%)	0.10%
L-Lysine	0.00%
Methionine+Cysteine	0.17%
Threonine	0.01%
Cr_2_O_3_	0.40%
Trace mineral premix^a^	0.20%
Vitamin premix^b^	0.04%
Additives	0.05%
**Total**	100.00%

a Supplied (per kg diet): Fe as FeSO_4_.7H_2_O, 100 mg; Mn as MnSO_4_.7H_2_O, 40 mg; Zn as ZnO, 80 mg; Cu as CuSO_4_.5H_2_O, 10 mg; Se as NaSeO_3_, 0.3 mg; and I as KI, 0.3 mg.

b Supplied (per kg diet): 19,000 IU vitamin A, 36.65 IU vitamin E, 386 IU vitamin D, 1.1 mg vitamin K (menadione dimethylpyrimidinoe bisulfate), 5 mg vitamin B_1_, 15 mg riboflavin, 25 mg niacin, 30 mg *d*-pantothenic acid, and 0.05 mg vitamin B_12_.

### Animal housing and tissue sampling

Pigs were housed individually in metabolic cages (0.7×1.5 m) with woven wire flooring in an environmentally controlled room (22 to 24°C) and were given ad libitum access to water through a water nipple. Through the 21 d experimental period, pigs were fed their assigned diets (CS or MS) four times per day (8: 00, 12: 00, 16: 00, and 20: 00) to ensure that all pigs received an ad libitum access to fresh diet. Pig weights and feed consumptions were determined daily throughout the duration of the trial. Blood samples were collected by venipuncture at 7: 00 on 14 d. At the end of the trial, pigs were euthanized with an intravenous injection of pentobarbital sodium (50 mg/kg body weight) and the liver samples were collected, weighted and stored at −80°C.

### Biochemical analysis

Liver and serum lipids were extracted and purified [21]. Triacylglycerol (TG) and cholesterol were measured as described previously [22]. Insulin, growth hormone (GH) and glucagon were measured with the use of electro-chemiluminescence immunoassays (Roche Diagnostics, Meylan, France). The activity of liver glucose-6-phosphate dehydrogenase (G6PD), fatty acid synthetase, acyl-CoA oxidase, and 3-hydroxy-3-methyl-glutaryl-CoA reductase (HMG-CoA reductase) were assayed according to methods as described elsewhere [23]. Dietary treatment effects were analyzed according to a completely randomized design using the general linear models (GLM) procedure of SAS (SAS Inc, Cary, NC, USA). Differences were considered to be significant with *P*<0.05.

### RNA extraction

Total RNA was isolated from liver using Trizol reagent (Invitrogen, Carlsbad, CA, USA) and further purified by Qiagen RNeasy Mini kit (Qiagen, Valencia, CA, USA). All the procedures were based on the manufacturer's protocol. The concentration of RNA was determined using spectrophotometry based on absorbance at 260 nm and integrity was monitored using the Agilent 2100 Bioanalyzer (Agilent Technologyies, USA).

### Microarrays

Agilent (Palo Alto, CA, USA) G2519F porcine gene expression microarray slides containing more than 40,000 probes were used for this experiment. Pooled samples were used to reduce the costs of the experiment. For pooling, equal amounts of total RNA from two different pigs were combined. Because there were eight pigs from each treatment group, four biological replicates were replicated on the microarrays. Cyanine-3 (Cy3) labeled cRNA was prepared from 0.5 ìg pooled RNA using the One-Color Low RNA Input Linear Amplification PLUS kit (Agilent) according to the manufacturer's instructions, followed by RNAeasy column purification (Qiagen, Valencia, CA). Dye incorporation and cRNA yield were checked with the NanoDrop ND-1000 Spectrophotometer. Microarrays were hybridized at 65°C for 17 h and washed with a Gene Expression Washing Buffer Kit (Agilent). Slides were scanned with an Agilent G2565BA microarray scanner.

### Microarray data collection and analysis

Microarray data were collected and analyzed using Agilent G2567AA Feature Extraction software, following Agilent's direct labeling protocol. The quantile method was used to normalize the probe intensities across the whole set of arrays [24]. Three criteria were used to determine statistically significant differential expression of genes in pigs between CS-fed and MS-fed group: (1) statistical significance: *P* value as determined by *t* test≤0.05; (2) reliability: a spot quality flag P (“P”, a quality flag assigned by the software package); (3) relevance: a minimal fold change between the means of the two groups>1.1. Raw data were deposited in the National Center for Biotechnology Information Gene Expression Omnibus in accordance with “minimum information about microarray experiments” guidelines (all data is MIAME compliant) and given accession numbers GSE20721.

### Real-time RT-PCR

Real-time PCR primers were designed (Takara, Dalian) to assay 12 differentially expressed genes (Table 2). The β-actin was used as the reference gene. Briefly, a total of 500 ng RNA was reversely transcribed using High capacity cDNA reverse Transcription Kit (Invitrogen) for each pig. Real-time RT-PCR reactions for 10 target genes and the housekeeping gene were performed using Applied Biosystems Power SYBR Green PCR Master Mix in a BioRad iCycler with minor modifications. Fluorescein was added at a final concentration of 10 nM as the reference dye. Cycling conditions were as follows: 95°C for 5 min, 45 cycles of 95°C for 30 s, appropriate annealing temperature (Table 2) for 30 s, 72°C for 30 s, followed by 72°C for 5 min, 95°C for 1 min, 55°C for 1 min, followed by a melt curve analysis of 80 cycles of 10 s at 55°C with a 0.5°C increase every cycle. Gene expression data from replicate samples were averaged and analyzed using the Pfaffl [25] method to between the cassava and maize cycle threshold values. Determination of statistical significance was done by ANOVA (SAS Inc, Cary, NC, USA).

**Table 2:** Primer sequences of genes selected for analysis by real-time RT-PCR.

Gene^a^	Accessionnumber	Forward primer	Reverse primer	Temp(°C)
*socs2*	NM_001097461	TTGATTAGAGATAGTTCGCACTC	CGCAGATTAGTTGGTCCAG	62.0
*hnf4a*	NM_001044571	CGCTCTATGGTGTTTAAGGATG	AGTGCCGAGGGATGATGTA	60.6
*mst4*	NM_001143727	CCAGAGAAACCCACCAAA	CAATGTCGCAAAGATCCAAA	59.5
*galp*	NM_213825	AGGAATCGGGAAATACTC	ATGCTACTCTGAAGAATCT	56.9
*hmgr*	NM_001122988	GAGTGGTCCCACAAATGAAG	CACGGTCCCGATCTCTATG	60.5
*acox1*	AK232470	TGACGGGAATGTGTATGAAA	CAGGTGCTTGTGGTAAGA	59.1
*fasn*	NM_001099930	GTGTGAGCAGTTCTGATG	AGCCTATCATGCTGTAGC	59.5
*cpt1a*	NM_001129805	ACAAGCCATAGTCTTAACGAAA	GCCAGTCCAGGATAACAAA	59.5
*fbp*	NM_213979	CACGCAGCCAAGTGAAGA	ATGTATGATGCACTGCCAGA	60.8
*cs*	NM_214276	CATGAAGGTGGCAATGTAAGT	TGCTGCAAAGGACAAGTAG	59.8
*ppara*	AK232864	CGTATCCTGCGTATGAAG	GTGTGAGCCTAAGAAGTT	58.5
*dgat*	NM_214051	ACCTACCGCGATCTCTAC	AGCTGGATGAGGAACAGCAT	59.6
*β-actin*	AY550069	TCTGGCACCACACCTTCT	TGATCTGGGTCATCTTCTCAC	56.5

a Gene abbreviations: *socs2*, suppressor of cytokine signaling 2; *hnf4a*, hepatocyte nuclear factor 4; *mst4*, serine/threonine protein kinase MST4; *galp*, galanin-like peptide; *hmgr*, 3-hydroxy-3-methylglutaryl-coenzyme A reductase; *acox1*, acyl-Coenzyme A oxidase 1; *fasn*, fatty acid synthase; *cpt1a*, carnitine palmitoyltransferase 1A; *fbp*, secreted folate binding protein; *cs*, citrate synthase; *ppara*, peroxisome proliferator-activated receptor alfha; *dgat*, diacylglycerol acyltransferase; *srebp1c*, sterol regulatory element-binding protein 1c.

### Pathway and network analysis

The SBC Analysis System (http://sas.ebioservice.com) was used to further interrogate the differentially expressed genes from the experiment. Gene abbreviations for the 648 genes exceeding the FDR threshold of *P*<0.05 and their fold changes were uploaded into the online analysis system to identify potential pathways or networks associated with dietary starch treatment. The most relevant signaling or metabolic pathways and networks were identified using the “pathway to gene” option.

## Results

### Growth performance, metabolites and hormones

Growth parameters, metabolites and hormone concentrations are reported in Table 3. We found no differences (*P*>0.05) in either weight gain or feed intake between the CS and MS group during the 21-d treatment period. There were no significant differences in serum glucose and TG concentrations (*P*>0.05) between the two groups. However, long-term intake of a high amylopectin ratio starch (CS) acutely increased the liver total fat content (8.91±0.62 vs. 5.38±0.33 g/100 g wet tissue, *P*<0.01). Furthermore, both the liver and serum cholesterol concentrations were significantly elevated in the CS group (*P*<0.05). We also measured the concentrations of several blood hormones—GH, insulin and glucagon—to assess the physiological basis for changes in serum metabolites (Table 3). The GH is a protein-based poly-peptide hormone capable of stimulating growth and cell reproduction and regeneration in humans and other animals. Both the insulin and glucagon are important hormones involved in carbohydrate metabolism. We found that the dietary amylose-amylopectin ratio did not alter the serum GH or glucagon concentrations (*P*>0.05). However, compared with the MS-fed group, the serum insulin concentration increased by 28.8% (*P*<0.05) in CS-fed group.

**Table 3:** Influences of dietary amylose-amylopectin ratio on growth performance, serum metabolite and hormone concentrations.

Parameter	Cassava starch	Maize starch
**Growth performance**		
Average daily gain (g/d)	378.9±21.3	386.7±28.2
Average daily intake (g/d)	492.7±29. 1	509.4±35.6
**Metabolites**		
Serum glucose (mmol/L)	6.83±0.48	6.40±0.34
Serum triglyceride (mmol/L)	0.54±0.02	0.47±0.03
Serum cholesterol (mmol/L)	2.04±0.13*	1.54±0.10
Liver total fat (*g/100* *g wet tissue*)	8.91±0.62**	5.38±0.33
Liver triglyceride (µmol/g)	69.12±7.11	58.34±6.32
Liver cholesterol (µmol/g)	3.42±0.25**	2.78±0.19
**Metabolic hormones**		
GH (ng/mL)	1.03±0.04	0.92±0.03
Insulin (pmol/L)	72.42±5.93*	56.24±5.17
Glucagon (pg/mL)	31.33±6.12	26.51±5.74
**Liver enzymes** **(U/mg protein)**		
Glucose-6-phosphate dehydrogenase	35.16±2.91	33.25±2.42
Fatty acid synthetase	28.56±2.74*	23.38±2.15
Acyl-CoA oxidase	3.61±0.42	4.46±0.35
HMG-CoA reductase	6.2±0.51*	4.9±0.38

Note:

* means *P*<0.05;

** means *P*<0.01.

### Liver metabolic enzymes

The activities of several key enzymes involved in lipid metabolism were measured (Table 3). Our results showed that the activity of G6PD was not affected by the dietary starch sources (*P*>0.05). The fatty acid synthetase plays a key role in fatty acid synthesis, while the HMG-CoA reductase is the rate-controlling enzyme of the mevalonate pathway that produces cholesterol and other isoprenoids. The activities of the two enzymes were both significantly elevated in CS group (*P*<0.05). However, CS-feeding significantly decreased the activity of acyl-CoA oxidase (the key enzyme of the fatty acid beta-oxidation pathway).

### Hepatic transcriptome analysis (Microarrays)

Microarrays were used to identify genes differentially expressed due to different dietary starch treatments. Analysis of gene expression data identified 648 differentially expressed genes at a nominal *P* value of 0.05. These genes were involved in a wide variety of physiological and biological events, such as the immune response, metabolic process and signal transduction. A large proportion of these genes were found to be associated with carbohydrate and lipid metabolism. Genes included in Table S1 (Supporting Information Table S1) were selected from the Kyoto Encyclopedia of Gene and Genomes (KEGG) pathways for its association with carbohydrate and lipid metabolism.

### Confirmation of microarray findings with real-time RT-PCR

Quantitative real-time RT-PCR assays designed for 12 genes were used to validate our results. The genes were selected based on their involvement in signal transduction pathway (*socs2* and *hnf4a*) or because they are important components of the lipid metabolic process (*hmgr*, *acox1*, *fasn*, *cpt1a*, *fbp*, *cs*, *ppara*, and *dgat*). Our results indicated that most of genes investigated had results congruent between microarray and RT-PCR assays (Table 4). There were higher transcript levels for *hmgr*, *fasn* and concomitant lower transcript levels for *acox1* in CS group than in MS group. However, MS feeding significantly elevated the transcription of *ppara* and *fbp* (*P*<0.05). The result for *galp* was likely a false positive from the microarray, in which the more sensitive real-time PCR assay indicated no statistical differential expression of this gene.

**Table 4:** Comparison of the microarray and real-time RT-PCR results.

Gene	Microarray results^a^		Real-time PCR results^b^		Regulation^c^
	P value^d^	Fold change	P value	Fold change	
*socs2*	#	1.30	*	1.05	+
*hnf4a*	#	1.08	#	1.22	-
*mst4*	*	2.07	*	1.78	+
*galp*	**	3.21	#	1.04	-
*hmgr*	**	1.35	**	1.62	-
*acox1*	**	1.12	*	1.32	+
*fasn*	**	1.11	**	1.09	-
*cpt1a*	#	1.11	#	1.06	-
*fbp*	#	1.05	*	1.29	+
*cs*	#	1.05	#	1.11	-
*ppara*	*	1.07	*	1.25	+
*dgat*	#	1.10	#	1.01	+

a Results based on hybridization of 8 microarrays using 8 pooled samples.

b Results based on 16 individual samples.

c The regulation was basis on the real MS/CS ratio from real-time PCR assay. (+) means the fraction (MS/CS) was more than 1 (up-regulation), whereas (−) means the fraction is under 1 (down-regulation).

d Note: ^*^means *P*<0.05; ^**^means *P*<0.01; ^#^means *P*>0.05.

### Pathway analysis

All the 648 differentially expressed genes were imported into the SBC Analysis System to identify pathways affected by different dietary starches. Nineteen pathways were identified that may be affected by dietary starch sources (*P*<0.05). These pathways involved cell signaling cascades (i.e. PPAR and insulin signaling), disease pathogenesis and drug metabolism. In addition, several traditional metabolic pathways such as the purine metabolism and TCA cycle were both affected (Table 5).

**Table 5:** Pathways containing significant numbers of differentially expressed genes^a^.

Pathway	Hits	Total	Percent	P value
Alanine and aspartate metabolism	2	13	15.38%	0.0389
Antigen processing and presentation	7	42	16.67%	0.0001
Arachidonic acid metabolism	5	30	16.67%	0.0008
Autoimmune thyroid disease	3	32	9.38%	0.0371
Calcium signaling pathway	6	71	8.45%	0.0057
Cardiac muscle contraction	5	32	15.63%	0.001
Cell adhesion molecules (CAMs)	8	57	14.04%	0.0001
Citrate cycle (TCA cycle)	2	17	11.76%	0.05
Drug metabolism - cytochrome P450	3	24	12.50%	0.0187
ErbB signaling pathway	3	32	9.38%	0.0371
Focal adhesion	8	73	10.96%	0.0003
Glutathione metabolism	3	22	13.64%	0.0152
Graft-versus-host disease	3	25	12.00%	0.0206
Hematopoietic cell lineage	4	48	8.33%	0.0238
Insulin signaling pathway	5	47	10.64%	0.0223
Leukocyte transendothelial migration	5	44	11.36%	0.0036
Long-term depression	4	33	12.12%	0.0074
Long-term potentiation	3	24	12.50%	0.0187
Metabolism of xenobiotics by cytochrome P450	3	21	14.29%	0.0136
mTOR signaling pathway	2	15	13.33%	0.0491
PPAR signaling pathway	5	48	10.42%	0.0238
Purine metabolism	6	44	13.64%	0.0006
Regulation of actin cytoskeleton	6	66	9.09%	0.0041
Type I diabetes mellitus	3	32	9.38%	0.0371
Type II diabetes mellitus	2	15	13.33%	0.0491

a Selected from KEGG pathway database (http://www.genome.jp/kegg/pathway.html).

## Discussion

In mammals the liver is the central player in whole body energy homeostasis. Upon consumption of excess carbohydrate, digestion yields simple sugars that are converted to pyruvate (glycolysis), which is either oxidized to provide energy or channeled into pathways for synthesis of fatty acids (lipogenesis) when energy is available [26]. The coordinated regulation of these processes allows the efficient utilization of dietary carbohydrates. Key enzymes of these pathways are tightly regulated by posttranslational and allosteric mechanisms triggered by changes in hormones and dietary nutrients [27]. In this study, we examined differential gene expression using microarrays and real-time PCR in the livers of weaned pigs fed with a CS- or MS-based diet. We found that the growth performance was not affected by dietary amylose and amylopectin ratio. However, long-term ingestion of a high amylopectin ratio starch (CS) tended to increase the hepatic lipogenesis and steroidogenesis via up-regulating several lipogenic genes, such as *fasn* and *hmgr*.

Starch is the one of the most important energy sources for human and other mammals consisting on polymers of amylose and amylopectin [13], [14], [18]. Starches with a high amount of amylose are hard to hydrolyze, whereas fully gelatinized amylopectin is easily digested, which serves as a source of rapidly digestible starch [17]. Therefore, the ratio of amylose to amylopectin affects starch digestibility and its metabolic responses [15], [18]. In the present study, CS contains 20% of amylose and 80% of amylopectin (20∶80), whereas MS contains 10% higher amylose (30∶70) than CS as determined in our preliminary experiment. The liver total fat content of pigs in CS-fed group was more than 8% (Table 3), which suggests a moderate FLD in this group. Additionally, we also observed that a long-term ingestion of CS significantly elevated liver cholesterol concentration (*P*<0.01), even though there is no significant difference in body weight gain and feed intake between the two groups. Furthermore, the serum TG and cholesterol concentrations in the CS group increased 14.9% and 32.4%, respectively. To our astonishment, the CS-feeding did not alter the serum glucose concentration (*P*>0.05). This could be attributed in part to the elevated serum insulin concentration in the CS group (Table 3) and the time point for blood collection. As we know, the small intestine is the major site for the terminal digestion of dietary starch, and the average retention time of digesta in small intestine is about 4 hour, thus the postprandial circulated glucose as well as other metabolites should changes periodically [14]. In this study, the blood samples were collected before the first meal in the morning. At this time point, the serum glucose concentration may resume to the fasting level. Our results suggested that the metabolic responses of weaned pigs were tightly regulated by dietary starch compositions, and long-term ingestion of a high amylopectin ratio starch is able to produce a prolonged rise in serum insulin concentration which may be detrimental to whole body insulin sensitivity in the long term.

Ingestion of carbohydrate results in elevated blood glucose which rapidly triggers insulin release from β cells of the endocrine pancreas. It has long been recognized that insulin induces the transcription of lipogenic enzymes in liver [27], [28]. Based on microarray analysis, we identified many differentially expressed genes that involved in carbohydrate and lipid metabolism. The *acox1* encoded protein (Acyl-Coenzyme A oxidase 1) is the first enzyme of the fatty acid β-oxidation pathway, and defects in this gene result in accumulation of very long chain fatty acids in the body [29]. In this study, the transcription of *acox1* was down-regulated in the CS group (Table 4). On the other hand, the lipogenic genes such as *fasn* and *hmgr* were both up-regulated. These results from microarrays correlated well with the measurements of enzyme activities involved in hepatic lipids metabolism (Table 3). Therefore, the altered transcription profile may directly contribute to the elevated liver TG and cholesterol concentrations in CS-fed pigs. Our results are also in good agreement with previous findings that a high-glycaemic food (carbohydrates that are digested quickly and release their energy rapidly) elevates insulin secretion and causes accumulation of fat in liver and plasma [9].

**Figure 1:**
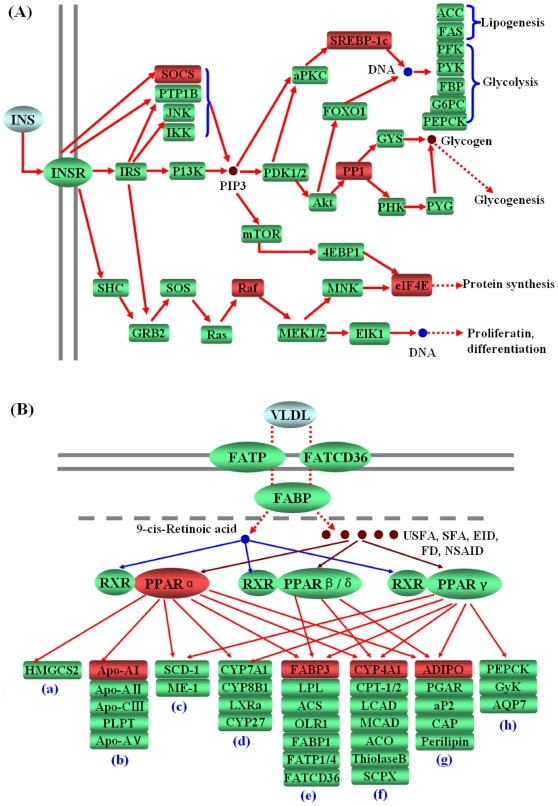
Influence of dietary amylose-amylopectin ratio on insulin (A) and PPAR (B) signaling pathways. (a) Ketogenesis, (b) Lipid transport, (c) Lipogenesis, (d) Cholesterol metabolism, (e) Fatty acid transport, (f) Fatty acid oxidation, (g) Adipocyte differentiation, (h) Gluconeogenesis; Gene symbols in red indicate genes that are differentially expressed.

With the SBC analysis system, we were able to identify biological pathways that appear to be affected by dietary amylose and amylopectin ratio. As we expected, several important pathways related to nutrient metabolism were found to be significantly affected (Table 5). It is a well known fact that the insulin and PPAR signaling pathways have profound effects on carbohydrate and lipid metabolism. Both of them were significantly affected by dietary starch sources (Fig. 1). Insulin drives glucose uptake in liver, muscle and fat cells, storing it as glycogen in the liver and muscle, and stopping use of fat as an energy source [30]. As shown in Fig. 1A, a number of genes involved in carbohydrate and lipid metabolism via insulin signaling pathway, and at least five important intermediate regulators (SOCS2, SREBP-1c, Raf, PP1 and eIF4E) were found to be differentially expressed in response to different dietary starches (*P*<0.05). Previous study indicated that the activation of genes responsible for lipogenesis in the liver by insulin is transcriptionally mediated by SREBP-1c, a membrane-bound transcription factor which can directly activate the expression of several genes (i.e. *fasn*) involved in the synthesis and uptake of cholesterol and fatty acids [31]. Compared with the MS group, the transcription of SREBP-1c was significantly elevated in CS group (*P*<0.05). Therefore, the elevated hepatic lipogenesis in pigs by CS-feeding may be attributed to the insulin-stimulated SREBP-1c gene transcription. This is also confirmed by mice models in which over-expression of SREBP-1c led to enhanced fatty acid synthesis rates and TG accumulation [32], whereas deletion of SREBP-1c exhibit diminished hepatic expression of lipogenic enzymes and a reduced fatty acid production [33]. Furthermore, SREBP-1c appears to be susceptible to diet since a high-glycaemic carbohydrate (fructose) acutely elevated its transcription [34]. Coupled with our present findings, the SREBP-1c can be a target for nutritional intervention during the process of steatosis and the slowly digestible carbohydrate (i.e. starches high in amylose) may help preventing or treating obesity and non-alcoholic fatty liver disease (NAFLD) in human.

Another group of transcription factors involved in the regulation of carbohydrate and lipid metabolism is the peroxisome proliferator-activated receptors (PPARs) [35]. Today, the role of PPARs in hepatic statosis has been investigated in detail [36], [37]. In this study, we found that the PPAR signaling pathway was also affected by dietary amylose and amylopectin ratio (Fig. 1B). The PPAR subfamily of nuclear receptors consists of members: PPARα, PPARβ/δ, and PPARγ, all of which play a role in energy homeostasis. PPARα and PPARβ/δ facilitate energy combustion, whereas PPARγ contribute to energy storage by enhancing adipogenesis [26], [38]. CS-feeding did not alter the transcriptional levels of *PPARG*, but significantly down-regulated the transcription of *PPARA* (*P*<0.05). As a result, the transcription of PPARα-regulated genes such as *FABP3* and *CYP4A1* were both declined (*P*<0.05). These results suggested that long-term ingestion of a rapidly digestible carbohydrate may elevate the accumulation of fat by transcriptional inhibiting of PPARα signaling pathway. Therefore, the PPARs can be another valuable target for nutritional intervention during the process of steatosis.

In summary, both the metabolic and transcriptomic responses of weaned pigs were tightly regulated by dietary starch compositions. And long-term ingestion of a rapidly digestible starch (high amylopectin ratio) significantly elevated the hepatic lipogenesis, which associated with a higher serum insulin concentration and more lipogenic genes expressed in liver. However, the slowly digestible starch may be more healthful for mammals as a long-term dietary energy source due to the transcriptional down-regulation of the lipogenesis and steroidogenesis.

## Supporting Information

Table S1 **Influence of dietary amylose-amylopectin ratio on hepatic expression of genes related to carbohydrates and lipids metabolism. (DOC)**
