# Supplementary material for: Metabolic and Transcriptomic Responses of Weaned Pigs Induced by Different Dietary Amylose and Amylopectin Ratio
Source: PLoS One. 2010 Nov 30;5(11):e15110. doi: 10.1371/journal.pone.0015110 (PMC2994909; doi:10.1371/journal.pone.0015110)
Supplement: Table S1 — Influence of dietary amylose-amylopectin ratio on hepatic expression of genes related to carbohydrates and lipids metabolism. (DOC) [file pone.0015110.s001.doc]

**Table S1.** Influence of dietary amylose-amylopectin ratio on hepatic expression of genes related to carbohydrates and lipids metabolisma

| **Gene** | **Description** | **Fold change^b^** | **P value** | **Gene** | **Description** | **Fold change** | **P value** |
| --- | --- | --- | --- | --- | --- | --- | --- |
| *acss2* | Acyl-CoA synthetase short-chain family 2 | 1.02 | 0.93 | *cyp11a1* | Cytochrome P450 11A1, mitochondrial | 1.07 | 0.80 |
| *alr1* | Aldehyde reductase | 1.08 | 0.43 | *cyp4a1* | Cytochrome P450 4A1 | 1.77 | 0.01 |
| *acl* | ATP citrate lyase | 1.08 | 0.79 | *cyp1a1* | Cytochrome P450 1A1 | 1.41 | 0.04 |
| *aco2* | Aconitase 2, mitochondrial | 1.04 | 0.39 | *cpt1a* | Carnitine palmitoyltransferase 1A, mitochondrial | 1.11 | 0.57 |
| *alr2* | Aldose reductase | 1.01 | 0.92 | *chat* | Choline acetyltransferase | 1.17 | 0.57 |
| *agppat4* | 1-acylglycerol-3-phosphate O-acyltransferase 4 | 1.11 | 0.53 | *cbr1* | Carbonyl reductase 1 | 1.11 | 0.50 |
| *asa* | Arylsulfatase A | 1.09 | 0.13 | *dlat* | Dihydrolipoamide S-acetyltransferase | 2.08 | 0.01 |
| *alox15* | Arachidonate 15-lipoxygenase | 1.05 | 0.59 | *dld* | Dihydrolipoamide dehydrogenase | 1.18 | 0.17 |
| *abat* | 4-aminobutyrate aminotransferase | 1.03 | 0.86 | *dlst* | Dihydrolipoamide S-succinyltransferase | 1.15 | 0.07 |
| *acadm* | Acyl-Coenzyme A dehydrogenase, C4 toC12 | 1.04 | 0.78 | *dgka* | Diacylglycerol kinase | 1.10 | 0.71 |
| *acox1* | Acyl-Coenzyme A oxidase 1, palmitoyl | 1.12 | 0.01 | *dgat* | Diacylglycerol acyltransferase | 1.10 | 0.71 |
| *acdal* | Acyl-Coenzyme A dehydrogenase, long chain | 1.06 | 0.06 | *fabp3* | Fatty acid binding protein 3 | 1.38 | 0.03 |
| *acads* | Acyl-Coenzyme A dehydrogenase, C2 to C3 | 1.06 | 0.79 | *fbp* | Fructose 1,6-bisphosphatase | 1.05 | 0.06 |
| *acl* | ATP citrate lyase | 1.32 | 0.45 | *fasn* | Fatty acid synthase | 1.11 | 0.01 |
| *acsl1* | Acyl coenzyme A synthetase long-chain 1 | 1.04 | 0.74 | *g6pc* | Glucose-6-phosphatase, catalytic subunit | 1.31 | 0.09 |
| *acat-2* | Sterol O-acyltransferase 2 | 1.82 | 0.43 | *gpi* | Glucose phosphate isomerase | 1.08 | 0.47 |
| *acat* | Acyl-coenzyme A: cholesterol acyltransferase | 1.70 | 0.04 | *gusb* | Beta-glucuronidase | 1.10 | 0.51 |
| *cyp51* | Cytochrome P450 51 | 1.03 | 0.80 | *galm* | Galactose mutarotase | 1.05 | 0.64 |
| *cyp27b1* | 25-hydroxyvitamin D3 1alpha-hydroxylase | 1.00 | 0.99 | *gulo* | Gulonolactone (L-) oxidase | 1.17 | 0.08 |
| *cyp39a1* | Cytochrome P450, family 39, subfamily A1 | 1.16 | 0.07 | *gsk3b* | Glycogen synthase kinase 3 beta | 1.11 | 0.14 |
| *cyp7* | Cholesterol 7alpha-hydroxylase | 1.06 | 0.90 | *gpat* | Glycerol-3-phosphate acyltransferase | 1.00 | 0.98 |
| *cyp8b1* | Cytochrome P-450 8B1 | 1.21 | 0.39 | *gnpat* | Glyceronephosphate O-acyltransferase | 1.07 | 0.24 |
| *crat* | Carnitine acetyltransferase, mitochondrial | 1.10 | 0.50 | *gba* | Glucosidase, beta; acid | 1.16 | 0.12 |
| *cds1* | CDP-diacylglycerol synthase | 1.22 | 0.03 | *gpx6* | Glutathione peroxidase 6 | 1.03 | 0.89 |
| *cad2* | Glutamate decarboxylase 2 | 1.11 | 0.82 | *gad1* | Glutamate decarboxylase 1 | 1.36 | 0.17 |
| *cs* | Citrate synthase, mitochondrial | 1.05 | 0.45 | *gltp* | Glycolipid transfer protein | 1.01 | 0.90 |
| *cry* | 3-hydroxyacyl-CoA dehydrogenase | 1.03 | 0.88 | *hmgr* | 3-hydroxy-3-methylglutaryl-coenzymeA reductase | 1.35 | 0.01 |

Table 4 (continued)

| **Gene** | **Description** | **Fold change** | **P value** | **Gene** | **Description** | **Fold change** | **P value** |
| --- | --- | --- | --- | --- | --- | --- | --- |
| *hsd11b2* | Hydroxysteroid (11-beta) dehydrogenase 2 | 1.70 | 0.55 | *pla2g2* | Phospholipase A2, group VII | 1.08 | 0.71 |
| *hk2* | Hexokinase 2 | 1.39 | 0.02 | *ptgs1* | Prostaglandin-endoperoxide synthase 1 | 1.36 | 0.13 |
| *had* | L-3-hydroxyacyl-CoA dehydrogenase | 1.12 | 0.20 | *plp1* | Proteolipid protein 1 | 1.04 | 0.81 |
| *hadha* | Hydroxyacyl-Coenzyme A dehydrogenase, alfa | 1.10 | 0.45 | *pccb* | Propionyl Coenzyme A carboxylase, beta | 1.01 | 0.89 |
| *itpk1* | Inositol 1,3,4-triphosphate 5/6 kinase | 2.09 | 0.01 | *pten* | Phosphatase and tensin homolog | 1.12 | 0.27 |
| *impa1* | Inositol (myo)-1(or 4)-monophosphatase 1 | 1.12 | 0.73 | *pik3cg* | Phosphoinositide-3-kinase, catalytic, gamma | 1.08 | 0.40 |
| *lalba* | Lactalbumin, alpha] | 1.20 | 0.47 | *pik3c3* | Phosphoinositide-3-kinase, class 3 | 1.01 | 0.83 |
| *lpl* | Lipoprotein lipase | 1.24 | 0.52 | *ppara* | Proxisome proliferator-activated receptor alfha | 1.07 | 0.04 |
| *lclat1* | Lysocardiolipin acyltransferase 1 | 1.20 | 0.06 | *pparg* | Peroxisome proliferator-activated receptor gamma | 1.07 | 0.56 |
| *lipa* | Lipase A | 1.22 | 0.07 | *rbp4* | Retinol binding protein 4, plasma | 1.01 | 0.95 |
| *lipe* | Lipase, hormone-sensitive | 1.06 | 0.59 | *sdhd* | Succinate dehydrogenase complex, subunit D | 1.01 | 0.73 |
| *ldhc* | Domestica lactate dehydrogenase-C | 2.09 | 0.18 | *suclg1* | Succinate-CoA ligase, alpha subunit | 1.05 | 0.03 |
| *ldha* | Domesticus lactate dehydrogenase-A | 1.14 | 0.24 | *sus2dd* | Dimeric dihydrodiol dehydrogenase | 1.00 | 0.99 |
| *ldhb* | Lactate dehydrogenase B | 1.07 | 0.66 | *sbab* | Cytochrome P450 21-hydroxylase | 1.22 | 0.21 |
| *ldlrap1* | Low-density lipoprotein adaptor protein | 1.02 | 0.71 | *st5ar2* | Steroid 5-alpha-reductase 2 | 1.54 | 0.07 |
| *ldlr* | Low density lipoprotein receptor | 1.03 | 0.80 | *sgms1* | Sphingomyelin synthase 1 | 1.10 | 0.32 |
| *lcthio* | Long-chain 3-ketoacyl-CoA thiolase | 2.05 | 0.02 | *sc4mol* | Sterol-C4-methyl oxidase-like | 1.08 | 0.09 |
| *mdh1* | Malate dehydrogenase 1 | 1.11 | 0.45 | *srebp2* | Sterol regulatory element binding transcription factor 2 | 1.06 | 0.43 |
| *mcee* | Methylmalonyl CoA epimerase | 1.02 | 0.68 | *srebp1c* | Sterol regulatory element-binding protein 1c | 1.28 | 0.02 |
| *mut* | Methylmalonyl Coenzyme A mutase | 1.08 | 0.57 | *slc2a11* | Solute carrier family 2, member 11 | 1.42 | 0.07 |
| *npl* | N-acetylneuraminate pyruvate lyase | 1.25 | 0.09 | *slc5a10* | Solute carrier family 5, member 10 | 1.56 | 0.13 |
| *oxct1* | 3-oxoacid CoA transferase 1 | 1.04 | 0.06 | *socs2* | Suppressor of cytokine signaling 2 | 1.30 | 0.06 |
| *pgk2* | Phosphoglycerate kinase 2 | 1.01 | 0.82 | *tpi1* | Triosephosphate isomerase 1 | 1.07 | 0.41 |
| *pck2* | Phosphoenolpyruvate carboxykinase 2 | 1.06 | 0.65 | *tkt* | Transketolase | 1.17 | 0.19 |
| *pc* | Pyruvate carboxylase, mitochondrial | 1.00 | 0.80 | *tbxas1* | Thromboxane A synthase 1 | 1.05 | 0.78 |
| *pnliprp1* | Pancreatic lipase-related protein 1 | 1.17 | 0.06 | *ugp2* | UDP-glucose pyrophosphorylase 2 | 1.05 | 0.56 |
| *ppap2c* | Phosphatidic acid phosphatase type 2C | 1.22 | 0.27 | *3bhsd* | 3-beta-hydroxysteroid dehydrogenase | 1.04 | 0.86 |

^a^ Genes were selected from the Kyoto Encyclopedia of Genes and Genomes pathways related to carbohydrate and lipid metabolism (<http://www.genome.jp/kegg/pathway.html>).

^b^ The fold change was basis on the ratio of CS/MS.
